# Non-HDL-C/HDL-C ratio is associated with carotid plaque stability in general population: A cross-sectional study

**DOI:** 10.3389/fneur.2022.875134

**Published:** 2022-09-15

**Authors:** Anran Wang, Yapeng Li, Lue Zhou, Kai Liu, Shaohua Li, Ce Zong, Bo Song, Yuan Gao, Yusheng Li, Chuansheng Tian, Yurong Xing, Yuming Xu, Longde Wang

**Affiliations:** ^1^Department of Neurology, The First Affiliated Hospital of Zhengzhou University, Zhengzhou, Henan, China; ^2^Chinese Preventive Medicine Association, Beijing, China; ^3^Physical Examination Center, The First Affiliated Hospital of Zhengzhou University, Zhengzhou, China; ^4^The General Office of Stroke Prevention Project Committee, National Health Commission of the People's Republic of China, Beijing, China

**Keywords:** lipid ratio, non-HDL-C/HDL-C, carotid plaque, stability, cross-sectional study

## Abstract

**Background:**

Carotid atherosclerosis, especially the rupture of unstable plaques, plays an important role in the development of stroke. A novel lipid ratio, the non-high-density lipoprotein cholesterol (non-HDL-C)/high-density lipoprotein cholesterol (HDL-C) ratio, contains both atherogenic and anti-atherogenic particle information, and has been shown to be associated with carotid atherosclerosis. However, there is no data on evaluating the association between non-HDL-C/HDL-C ratio and carotid plaque stability.

**Methods:**

This study was carried out on 27,436 urban workers aged 20 years or older who participated in a comprehensive health screening between January 2016 and December 2017. Carotid plaque stability was assessed using ultrasonography. Multinomial logistic regression models were used to explore the relationship between the non-HDL-C/HDL-C ratio and carotid plaque stability by odds ratios (*ORs*) and 95% confidence intervals (*CIs*). Subgroup and sensitivity analyses were performed to verify the robustness of the results.

**Results:**

Carotid plaque was detected in 7,161 (26.1%) participants, with stable and unstable plaque accounting for 3,277 (11.9%) and 3,884 (14.2%), respectively. The prevalence of stable carotid plaque substantially increased with increasing non-HDL-C/HDL-C ratio quartile levels (*p* for trend < 0.001) and with a similar association for unstable carotid plaque (*p* for trend < 0.001). The mean non-HDL-C/HDL-C ratios (mean ± *SD*) of non-carotid plaque (2.9 ± 1.1), stable carotid plaque (3.2 ± 1.2), and unstable carotid plaque (3.4 ± 1.4) gradually increased (*p* < 0.001). In multinomial logistic regression, *ORs* (95% *CIs*) for the highest vs. lowest quartile of the non-HDL-C/HDL-C ratio were 1.70 (1.48–1.95) between stable carotid plaques and no carotid plaque, 2.34 (2.06–2.67) between unstable carotid plaques and no carotid plaque, and 1.38 (1.18–1.61) between unstable carotid plaques and stable carotid plaque, after adjusting for common cardiovascular risk factors. The results of subgroup analysis and sensitivity analysis were similar.

**Conclusion:**

Our findings suggested that the non-HDL-C/HDL-C ratio was significantly associated with carotid plaque stability and might be a useful indicator for the early identification of high-risk carotid plaque.

## Introduction

Stroke is a leading cause of death and disability worldwide (1). Carotid plaque stability plays a fundamental role in the development of ischemic stroke (2, 3). Rupture of unstable carotid plaque can lead to thrombosis, resulting in cerebrovascular occlusion and infarction. Approximately, 18–25% of ischemic stroke thromboembolisms originate from ruptured carotid plaques (4), and carotid plaque is also an important cause of cryptogenic stroke (5). However, carotid ultrasound screening in the general population is not recommended in the current guideline (6). Therefore, early identification of carotid plaque stability could help discover people at high risk of stroke, who might benefit from early pharmacological or surgical intervention.

It is well-known that high-density lipoprotein cholesterol (HDL-C) and non-HDL cholesterol (non-HDL-C) are associated with atherosclerosis (7–9). Non-HDL-C is considered to be a key factor underlying the process contributing to cardiovascular disease and atherosclerosis (10). The National Lipid Association has identified non-HDL-C as a primary therapeutic target (11), HDL-C, which is composed of the smallest and densest lipoprotein particles, inhibits atherosclerosis (12). HDL-C is negatively associated with cardiovascular (CV) events with each 1 mg/dl increase reducing CV events by 2–3% (7, 13, 14), and it exerts cardiovascular protective effects mainly through reverse cholesterol transfer, anti-inflammatory, antioxidant, anti-apoptotic, and vasodilatory effects (14). A novel lipid ratio, the non-HDL-C/HDL-C ratio, contains both atherogenic and anti-atherogenic particles information, and has been shown to be associated with a variety of dyslipidemia-related diseases such as diabetes mellitus (15–17), liver disease (18, 19), metabolic syndrome (20), and previous studies also demonstrated that the non-HDL-C/HDL-C ratio was associated with carotid atherosclerosis (21–23). In addition, the Atherosclerosis Risk in Communities (ARIC) study even found an independent association between non-HDL-C/HDL-C ratio and carotid plaque lipid core (24). The thicker the lipid core, the more likely it is to cause expansion of the necrotic core of the plaque, resulting in plaque rupture (25). Moreover, recently studies showed that the non-HDL-C/HDL-C ratio might be a better predictor of cardiovascular events than traditional lipid indices (26, 27). However, to date, there is no data on evaluating the association between non-HDL-C/HDL-C ratio and carotid plaque stability.

Therefore, we performed this study to clarify whether non-HDL-C/HDL-C ratio is significantly associated with carotid plaque stability.

## Methods

### Participants

The study population consisted of 28,537 Chinese adults who participated in a health examination in stroke screening sites of the First Affiliated Hospital of Zhengzhou University from January 2016 to December 2017. The exclusion criteria of this study were: subjects with any history of malignancy, infectious diseases, acute inflammation, liver disease, or renal disease. We also excluded subjects with missing data on TC or fasting blood glucose (FBG). After applying our exclusion criteria, a total of 27,436 participants were enrolled in this study.

### Data collection

We collected individual sociodemographic information (e.g., sex, age, and education), history of chronic diseases (e.g., diabetes, dyslipidemia, hypertension, coronary heart disease, and stroke), and lifestyle factors (e.g., smoking, drinking, vegetable and fruit consumption, physical activity, etc.) *via* a standard questionnaire by trained interviewers. Definition of history of stroke and coronary artery disease: previously diagnosed by a medical specialist or provided imaging data to support the diagnosis. Smoking, defined as smoking 1 cigarette per day for more than 1 year. Drinking, defined as alcoholic drink of at least ≥45 g each time per day during the last year. Vegetable consumption and Fruit consumption were divided into two groups (≥5 days/week and <5 days/week) using a standard consumption of 200 g per day. Physical activity, defined as regular exercise for at least 30 min per time in no less than 3 times per week. We measured weight, height, and resting blood pressure such as systolic blood pressure (SBP) and diastolic blood pressure (DBP). Obesity was defined as BMI ≥ 28 kg/m^2^.

In addition, overnight fasting blood samples were obtained from all subjects. Fasting blood glucose was measured using the glucose oxidase method. Lipid levels including total cholesterol (TC), triglyceride (TG), HDL-C, and low-density lipoprotein cholesterol (LDL-C) were measured using Olympus Au5400 automated biochemistry analyzer (First Chemical Co, Ltd, Japan). Hypertension was defined as SBP and/or DBP ≥ 140/90 mmHg, or the usage of antihypertensive medications. Dyslipidemia was defined as TG ≥ 2.26 mmol/L, TC ≥ 6.22 mmol/L, HDL-C < 1.04 mmol/L, LDL-C ≥ 4.14 mmol/L, self-reported history, or taking lipid-lowering drugs. Diabetes mellitus (DM) was defined as self-reported history, FPG ≥ 7.0 mmol/L, or taking antidiabetic agents.

### Assessment of carotid plaque stability

Ultrasound technologists evaluated carotid plaques by qualified sonographers using the iU22 (Philips Healthcare), HA500 (Hitachi Healthcare), and DC-8 (Mindray), ultrasound system with 5–10 MHz transmission frequency. Two qualified sonographers measured each participant separately; discrepancies in measurement data were resolved by consensus. We examined plaques of bilateral common carotid artery, internal carotid artery, external carotid artery, and bulb. Carotid plaque was defined as a focal structure encroaching into the arterial lumen by at least 0.5 mm or 50% of the surrounding CIMT value, or CIMT > 1.5 mm (28, 29). Stable carotid plaques had a high level of homogeneous echogenicity and homogeneous texture with a regular smooth morphology. Unstable carotid plaques had an incomplete fibrous cap or ulceration with low level or heterogeneous echogenicity (30).

### Statistical analysis

The population was divided into four groups based on the quartiles of the non-HDL-C/HDL-C ratio. Categorical variables were presented as frequency (%), which was compared using chi-square analysis. Continuous variables were described as the median with an interquartile range owing to the skewed distribution, which were compared by variance (ANOVA) or Mann–Whitney *U*-tests for continuous variables.

When participants were divided into 3 groups according to carotid plaque stability (non-carotid plaque, stable carotid plaque, and unstable carotid plaque), multinomial logistic regression models were used to explore the relationship between the non-HDL-C/HDL-C ratio and carotid plaque stability. To adjust for potential confounders, two models were developed: Model 1, adjusted for age, sex, education, smoking status, drinking status, vegetable consumption, fruit consumption, physical activity, BMI ≥ 28 kg/m^2^ (yes or no), stroke, coronary heart disease, hypertension, antihypertensive agents, diabetes mellitus, antidiabetic agents, lipid-lowering agents; Model 2, further adjusted for TG and FBG. The results were presented as odds ratios (*ORs*) and 95% confidence intervals (*CIs*). To verify the robustness of the relationship between non-HDL-C/HDL-C ratio and carotid plaque stability, analyses were carried out for different subgroups. In view of the effect of lipid-lowering agents on non-HDL-C/HDL-C ratio and carotid plaque stability, sensitivity analysis was performed after excluding people taking lipid-lowering agents.

All analyses above were conducted using R software (version 3.6.3). A two-sided *p* < 0.05 was considered statistically significant.

## Results

### Baseline characteristics

A total of 27,436 participants were recruited, 12,866 (46.9%) of them were male. The median (IQR) age of overall participants was 48 (41–55) years, and the median non-HDL-C/HDL-C ratio was 2.82 (*IQR*: 2.16–3.60). Compared with participants in the lowest quartile of non-HDL-C/HDL-C ratio, those with higher non-HDL-C/HDL-C ratio were more likely to be older and male; to be smoking, drinking, vegetable, and fruit consumption, active physical activity, obesity; to have a higher prevalence of hypertension, diabetes, and dyslipidemia; to have higher use of antidiabetic, antihypertensive, and lipid-lowering agents use, to have a higher level of SBP, DBP, FBG, TC, TG, LDL-C, and non-HDL level, while more likely to have a lower level of HDL-C. The characteristics of participants according to quartiles of the non-HDL-C/HDL-C ratio are presented in Table 1.

**Table 1:** Baseline characteristics of the study participants.

**Characteristics**	**Overall**	**Quartiles of the Non-HDL-C/HDL-C ratio**				***P*-value^†^**
		**Q1 (<2.16)**	**Q2 (2.16–2.82)**	**Q3 (2.82–3.60)**	**Q4 (>3.60)**	
No. of patients	27,436	6,858	6,857	6,863	6,858	
Age, years	48.0 (41.0–55.0)	44.0 (39.0–52.0)	48.0 (41.0–55.0)	49.0 (43.0–56.0)	50.0 (44.0–57.0)	<0.001
Male, sex	12,866 (46.9)	1,812 (26.4)	2,785 (40.6)	3,641 (53.1)	4,628 (67.5)	<0.001
High school or above, *n* (%)	17,032 (62.1)	4,819 (70.3)	4,367(63.7)	4,046 (59.0)	3,800 (55.4)	<0.001
Smoking, *n* (%)	6,548 (23.9)	803 (11.7)	1,335 (19.5)	1,785 (26.0)	2,625 (38.3)	<0.001
Drinking, *n* (%)	5,401 (19.7)	690 (10.1)	1,165 (17.0)	1,547 (22.5)	1,999 (29.1)	<0.001
Vegetable (<5d/w), *n* (%)	12,940 (47.2)	2,692 (43.3)	2,878 (46.3)	2,982 (47.9)	3,060 (49.2)	<0.001
Fruit (<5d/w), *n* (%)	22,734 (82.9)	5,555 (81.0)	5,586 (81.5)	5,747 (83.7)	5,844 (85.2)	<0.001
Active physical activity, n (%)	19,798 (72.2)	4,805 (70.1)	5,046 (73.6)	5,005 (72.9)	4,942 (72.1)	<0.001
BMI ≥ 28 (kg/m^2^)	4,595 (16.7)	457 (6.7)	869 (12.7)	1,301 (19.0)	1,968 (28.7)	<0.001
Hypertension, *n* (%)	11,236 (41.0)	1,992 (29.0)	2,510 (36.6)	3,090 (45.0)	3,644 (53.1)	<0.001
Diabetes, *n* (%)	2,914 (10.6)	389 (5.7)	553 (8.1)	746 (10.9)	1,226 (17.9)	<0.001
Dyslipidemia, *n* (%)	10,403 (37.9)	370 (5.4)	1,226 (17.9)	2,997 (43.7)	5,810 (84.7)	<0.001
Antihypertensive agents, *n* (%)	3,580 (13.0)	615 (9.0)	790 (11.5)	973 (14.2)	1,202 (17.5)	<0.001
Antidiabetic agents, *n* (%)	1,305 (4.8)	191 (2.8)	273 (4.0)	348 (5.1)	493 (7.2)	<0.001
Lipid-lowering agents, *n* (%)	731 (2.7)	110 (1.6)	162 (2.4)	165 (2.4)	294 (4.3)	<0.001
Stroke, *n* (%)	487 (1.8)	129 (1.9)	108 (1.6)	123 (1.8)	127 (1.9)	0.520
Coronary heart disease, *n* (%)	617 (2.2)	151 (2.2)	146 (2.1)	144 (2.1)	176 (2.6)	0.224
Carotid plaque						<0.001
No carotid plaque, *n* (%)	20,275 (73.9)	5,768 (84.1)	5,238 (76.4)	4,936 (71.9)	4,333 (63.2)	
Stable carotid plaque, *n* (%)	3,277 (11.9)	564 (8.2)	748 (10.9)	869 (12.7)	1,096 (16.0)	
Unstable carotid plaque, *n* (%)	3,884 (14.2)	526 (7.7)	871 (12.7)	1,058 (15.4)	1,429 (20.8)	
DBP, mm Hg	81.0 (73.0–90.0)	77.0 (70.0–85.0)	80.0 (72.0–89.0)	83.0 (75.0–91.0)	85.0 (77.0–94.0)	<0.001
Fasting blood glucose, mmol/L	5.20 (4.80–5.70)	5.00 (4.70–5.40)	5.10 (4.75–5.60)	5.20 (4.80–5.70)	5.40 (4.90–6.10)	<0.001
Total cholesterol, mmol/L	4.62 (4.07–5.24)	4.06 (3.62–4.52)	4.46 (4.00–4.96)	4.76 (4.29–5.29)	5.27 (4.74–5.88)	<0.001
Triglyceride, mmol/L	1.26 (0.89–1.86)	0.82 (0.66–1.04)	1.10 (0.87–1.43)	1.43 (1.11–1.90)	2.11 (1.58–2.96)	<0.001
HDL cholesterol, mmol/L	1.20 (1.03–1.41)	1.48 (1.31–1.67)	1.28 (1.14–1.43)	1.14 (1.02–1.27)	0.98 (0.88–1.10)	<0.001
LDL cholesterol, mmol/L	2.70 (2.21–3.23)	2.16 (1.84–2.51)	2.66 (2.28–3.03)	2.94 (2.51–3.38)	3.21 (2.66–3.76)	<0.001
Non-HDL cholesterol, (mmol/l)	3.38 (2.82–4.00)	2.57 (2.24–2.91)	3.18 (2.84–3.56)	3.61 (3.25–4.04)	4.28 (3.82–4.80)	<0.001

BMI, body mass index; Continuous variables are expressed as median (interquartile range). Categorical variables are expressed as frequencies (percentage);

† *P*-values were derived from Mann–Whitney *U*-tests for continuous variables, and Chi-square tests for categorical variables.

### Non-HDL-C/HDL-C ratio and carotid plaque stability

**Figure 1:**
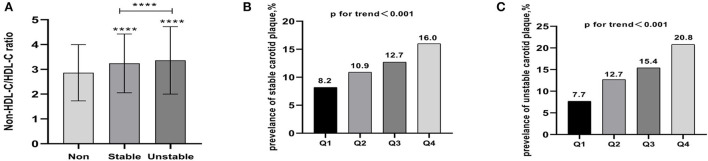
**(A)** The mean non-HDL-C /HDL-C ratios of non-carotid plaque, stable carotid plaque, and unstable carotid plaque (mean ± *SD*) increased gradually and this trend was statistically significant. **(B)** Prevalence of stable carotid plaque stratified by quartile the non-HDL-C/HDL-C ratio. **(C)** Prevalence of unstable carotid plaque stratified by quartile the non-HDL-C/HDL-C ratio. **** *p* < 0.001.

Carotid plaque was detected in 7,161 (26.1%) respondents, with stable and unstable plaque accounting for 3,277 (11.9%) and 3,884 (14.2%), respectively. As non-HDL-C/HDL-C ratio levels increased from the lowest quartile to the highest quartile, the prevalence was increased from 8.2 to 16% for stable carotid plaque (*p* for trend < 0.001) and from 7.7 to 20.8% for unstable carotid plaque (*p* for trend < 0.001). The mean non-HDL-C/HDL-C ratios of non-carotid plaque (mean ± *SD*, 2.9 ± 1.1), stable carotid plaque (mean ± *SD*, 3.2 ± 1.2), and unstable carotid plaque (mean ± *SD*, 3.4 ± 1.4) increased gradually and this trend was statistically significant (*p* < 0.001; Figure 1).

In multinomial logistic regression model 1 compared stable carotid plaques with no carotid plaque, the *ORs* (95% *CIs*) for the highest quartile of the non-HDL-C/HDL-C ratios were 1.71 (1.51–1.94). For every 1 unit increase in the non-HDL-C/HDL-C ratio, the prevalence of stable carotid plaque increased by 1.17 times. Compared unstable carotid plaques with no carotid plaque, the *ORs* (95% *CIs*) for the highest quartile of the non-HDL-C/HDL-C ratios were 2.21 (1.96–2.49). Moreover, for every 1 unit increase in the non-HDL-C/HDL-C ratio, the prevalence of unstable carotid plaque increased by 1.21 times. Compared to unstable carotid plaques with stable carotid plaque, the *ORs* (95% *CIs*) for the highest quartile of the non-HDL-C/HDL-C ratios were 1.29 (1.12–1.50). Moreover, for every 1 unit increase in the non-HDL-C/HDL-C ratio, the prevalence of unstable carotid plaque increased by 1.04 times.

Further adjustment for TG and FBG in model 2 did not change the association between the non-HDL-C/HDL-C ratio and carotid plaque stability. The corresponding *ORs* (95% *CIs*) for the highest vs. lowest quartile of the non-HDL-C/HDL-C ratio were 1.70 (1.48–1.95) between stable carotid plaques and no carotid plaque, 2.34 (2.06–2.67) between unstable carotid plaques and no carotid plaque, and 1.38 (1.18–1.61) between unstable carotid plaques and stable carotid plaque (Table 2).

**Table 2:** Multinomial logistic odds ratios (*ORs*) (95% *CI*) of the association of the non-HDL-C/HDL-C ratio with carotid stability.

**Outcomes**	**Quartiles of the Non-HDL-C/HDL-C ratio**				***P*-trend**	**Per 1**
	**Q1 (<2.16)**	**Q2 (2.16–2.82)**	**Q3 (2.82–3.60)**	**Q4 (>3.60)**		**unit increase**
Stable vs. non-carotid plaque				
Crude model	Reference	1.45 (1.28–1.65)	1.73 (1.52-1.95)	2.37 (2.10-2.67)	<0.001	1.28 (1.24-1.33)
Model 1	Reference	1.24 (1.09–1.42)	1.29 (1.13-1.47)	1.71 (1.51-1.94)	<0.001	1.17 (1.13-1.21)
Model 2	Reference	1.24 (1.09–1.41)	1.29 (1.13-1.47)	1.70 (1.48-1.95)	<0.001	1.21 (1.15-1.27)
Unstable vs. non-carotid plaque				
Crude model	Reference	1.86 (1.64–2.12)	2.32 (2.04–2.63)	3.43 (3.04–3.88)	<0.001	1.36 (1.32–1.41)
Model 1	Reference	1.51 (1.33–1.71)	1.62 (1.44–1.83)	2.21 (1.96–2.49)	<0.001	1.21 (1.18–1.25)
Model 2	Reference	1.52 (1.35–1.73)	1.66 (1.47–1.88)	2.34 (2.06–2.67)	<0.001	1.35 (1.30–1.41)
Unstable vs. stable carotid plaque				
Crude model	Reference	1.29 (1.08–1.53)	1.34 (1.14–1.59)	1.45 (1.24–1.70)	<0.001	1.06 (1.03–1.10)
Model 1	Reference	1.22 (1.04–1.42)	1.26 (1.08–1.46)	1.29 (1.12–1.50)	0.002	1.04 (1.00–1.08)
Model 2	Reference	1.23 (1.05–1.44)	1.29 (1.11–1.51)	1.38(1.18–1.61)	<0.001	1.12 (1.07–1.18)

**Model 1:** adjusted for age, sex, education, smoking status, drinking status, vegetable consumption, fruit consumption, physical activity, BMI ≥ 28 kg/m^2^ (yes or no), stroke, coronary heart disease, hypertension, antihypertensive agents, diabetes mellitus, antidiabetic agents, lipid-lowering agents.

**Model 2:** further adjusted for Triglyceride, Fasting blood glucose.

### Subgroup analysis and sensitivity analysis

**Figure 2:**
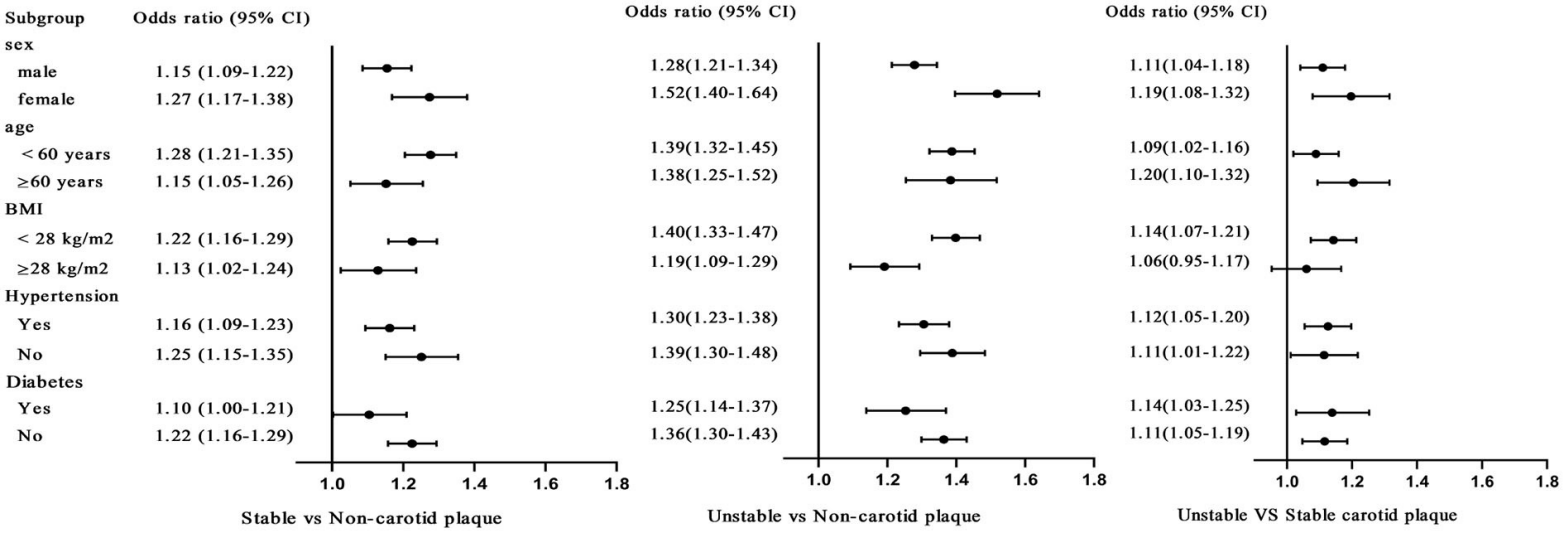
Subgroup analysis was conducted by stratification according to sex, age, BMI, hypertension, and diabetes, when the non-HDL-C/HDL-C ratio was considered as a continuous variable. After the fully adjustment, the same variables as Model 3 in Table 2, the odds ratios (*ORs*; 95% *CIs*) of the non-HDL-C/HDL-C ratio and carotid stability.

When the non-HDL-C/HDL-C ratio was considered as a continuous variable, subgroup analysis was conducted by stratification according to sex, age, BMI, hypertension, and diabetes. After the full adjustment variables, the association between non-HDL-C/HDL-C ratio and carotid plaque stability remained significant in gender, age, hypertension, and diabetes subgroups. While in a subgroup analysis of BMI ≥ 28 (kg/m^2^), there was no statistical significance between unstable carotid plaques and no carotid plaque (*OR* 1.06, 95% *CI* 0.95–1.17; Figure 2).

This sensitivity analysis was performed to assess the relationship between non-HDL-C/HDL-C ratio and carotid plaque stability in the subsample without taking lipid-lowering agents. After multivariable adjustment for the risk factors, sensitivity analyses showed similar results (Table 3).

**Table 3:** Sensitivity analysis^†^.

**Outcomes**	**Quartiles of the Non-HDL-C/HDL-C ratio index**				***P*-trend**	**Per 1**
	**Q1 (<2.16)**	**Q2 (2.16–2.82)**	**Q3 (2.82–3.60)**	**Q4 (>3.60)**		**Unit increase**
Stable vs. no carotid plaque				
Crude model	Reference	1.45 (1.28–1.63)	1.81 (1.61–2.03)	2.64 (2.36–2.96)	<0.001	1.28 (1.24–1.33)
Model 1	Reference	1.23 (1.08–1.41)	1.28 (1.12–1.46)	1.73 (1.52–1.97)	<0.001	1.17 (1.13–1.21)
Model 2	Reference	1.23 (1.07–1.41)	1.27 (1.11–1.45)	1.70 (1.48–1.96)	<0.001	1.21 (1.15–1.27)
Unstable vs. no carotid plaque				
Crude model	Reference	1.82 (1.62–2.04)	2.40 (2.15–2.69)	3.61 (3.24–4.03)	<0.001	1.36 (1.32–1.41)
Model 1	Reference	1.50 (1.32–1.71)	1.64 (1.45–1.86)	2.18 (1.93–2.46)	<0.001	1.21 (1.18–1.25)
Model 2	Reference	1.51 (1.33–1.72)	1.67 (1.48–1.89)	2.28 (2.00–2.60)	<0.001	1.35 (1.30–1.41)
Unstable vs. stable carotid plaque				
Crude model	Reference	1.25 (1.07–1.47)	1.33 (1.14–1.55)	1.37 (1.18–1.58)	<0.001	1.06 (1.03–1.10)
Model 1	Reference	1.22 (1.04–1.43)	1.29 (1.10–1.50)	1.26 (1.08–1.47)	0.002	1.04 (1.00–1.08)
Model 2	Reference	1.23 (1.05–1.45)	1.32 (1.13–1.54)	1.34 (1.14–1.58)	<0.001	1.12 (1.07–1.18)

Multinomial logistic *ORs* (95% *CI*) of the association of the non-HDL-C/HDL-C ratio with carotid plaque and its stability.

Model 1: adjusted for age, sex, education, smoking status, drinking status, vegetable consumption, fruit consumption, physical activity, BMI ≥ 28 kg/m^2^ (yes or no), stroke, coronary heart disease, hypertension, antihypertensive agents, diabetes mellitus, antidiabetic agents, lipid-lowering agents.

Model 2: further adjusted for Triglyceride, Fasting blood glucose.

† Sensitivity analysis was performed in participants without taking lipid-lowering agents.

## Discussion

This is the first population-based study to explore the relationship between non-HDL-C/HDL-C ratio and carotid plaque stability. We found that the prevalence of both stable and unstable carotid plaque increased significantly with increasing non-HDL-C/HDL-C ratios, independent of other relevant factors. Meanwhile, the mean non-HDL-C /HDL-C ratio gradually increased for non-carotid plaques, stable carotid plaques, and unstable carotid plaques; and this trend was statistically significant. Our findings, for the first time, demonstrated that non-HDL-C /HDL-C ratio was associated with carotid plaque stability in the general population.

Early atherosclerosis occurs mainly in the peripheral vasculature, such as the femoral and carotid arteries (31). The burden of carotid atherosclerosis has been increasing. A systematic review revealed the global burden of carotid atherosclerotic disease, with 21.1% of people aged 30–79 years suffering from carotid plaque in 2020, equivalent to 815.76 million people (32), and it was known that unstable plaque was an important factor in the development of cardiovascular disease (33). Moreover, many studies had shown that early preventive treatment and risk factor intervention were beneficial (34–36). However, carotid ultrasound screening in the general population is not recommended in the current guideline (6). Therefore, simple and accessible biomarkers for early determination of carotid plaque stability can help improve the understanding of the pathophysiology of cardiovascular disease and identify high-risk patients who may benefit from early intervention.

Our study found a gradual increase in the prevalence of carotid plaque as the non-HDL-C/HDL-C ratio gradually increased. As with our findings, the previous studies had shown that the non-HDL-C/HDL-C ratio was associated with carotid atherosclerosis (21–23). Qin et al. (21) found that carotid intima-media thickness gradually increased in the quartile of non-HDL-CHDL-C ratio in Chinese individuals of metabolic syndrome (*p* trend < 0.05). A multicenter study (22) found that postmenopausal women with higher non-HDL-C/HDL-C ratios had a greater chance of developing carotid atherosclerotic plaque (*OR*: 1.30, 95% *CI*: 1.07–1.58, *p* = 0.009) when adjusted for other cardiovascular risk factors. Recently, studies found that the non-HDL-C/HDL-C ratio might be a more accurate predictor of cardiovascular disease (26, 27). In addition, an asymptomatic polyvascular abnormalities in Community study (37) found that the odds of unstable carotid plaques at non-HDL-C levels in the middle and highest trilaterals were 1.02 (95% *CI*, 0.84–1.23) and 1.50 (95% *CI*, 1.23–1.82), respectively, after adjusting for confounders. Moreover, ARIC (24) study even found an independent association between non-HDL-C/HDL-C ratio and carotid plaque lipid core. It is well-known that plaque rich in lipid core is unstable and easy to rupture (25). All the above studies indirectly supported our findings that the non-HDL-C/HDL-C ratio might be associated with carotid plaque stability.

As a clinically easily accessible biomarker, the non-HDL-C/HDL-C ratio collects information on all atherogenic and antiatherogenic lipid particles. Non-high-density lipoprotein cholesterol (non-HDL-C) level is calculated by subtracting high-density lipoprotein cholesterol (HDL-C) from TC. Non-HDL-C consists of LDL-C, very low-density lipoprotein (VLDL-C), intermediate-density lipoprotein (IDL-C), chylomicrons, and their TG-rich lipoprotein remnants, and the protein mainly contains apolipoprotein B, which is a strong indicator of atherogenicity (13, 38). Non-HDL-C is considered to be a key factor underlying the process contributing to most cardiovascular diseases (10). The National Lipid Association has identified non-HDL-C as a primary therapeutic target (11), and the ESC/EAS guidelines for the management of dyslipidemia also recommended the inclusion of non-HDL in the assessment of cardiovascular disease risk (39). HDL-C is composed of the smallest and densest lipoprotein particles and mainly contains apolipoprotein A-I (APOA-I), which inhibits the production and mobilization of inflammatory cells and promotes the reversal of cholesterol transport (RCT) to inhibit atherosclerosis (12). HDL-C is thought to be negatively associated with cardiovascular disease events (7). The non-HDL-C/HDL-C ratio can reflect the balance between atherogenic and anti-atherogenic lipid particles, which may be the underlying mechanism for its relationship with plaque stability.

There are several limitations in our study. First, this is a cross-sectional study and no conclusions can be drawn on the causal relationship between non-HDL-C/HDL-C ratio and carotid plaque stability. Second, carotid plaque stability is assessed using ultrasound, which is not as accurate as MRI or angiography and cannot be verified with pathological specimens. However, we corrected for bias with a two-person blinded assessment. Third, our study did not collect information on HDL functionality, which is a better predictor of CV risk than HDL-C levels (40, 41), and it may be useful for high-risk carotid plaque identification. Finally, we did not collect follow-up information, which limited our ability to prospectively study, the impact of baseline non-HDL-C/HDL-C ratio on the evolution of plaque stability and cardiovascular events. Therefore, future prospective cohort studies are clearly needed.

## Conclusions

In conclusion, this study found that the non-HDL-C/HDL-C ratio was associated with carotid plaque stability in the general population. The non-HDL-C/HDL-C ratio was highest in those with unstable carotid plaque, followed by those with stable carotid plaque and lowest in those with no carotid plaque. Our findings suggest that an elevated non-HDL-C/HDL-C ratio is independently associated with carotid plaque and its stability.

## Data availability statement

The original contributions presented in the study are included in the article/supplementary material, further inquiries can be directed to the corresponding author/s.

## Ethics statement

The studies involving human participants were reviewed and approved by the Ethics Committee of the First Affiliated Hospital of Zhengzhou University. Written informed consent to participate in this study was provided by the patient/participants legal guardian/next of kin.

## Author contributions

YMX and LW designed the research. YPL, LZ, YG, KL, SL, CZ, and YRX helped with the acquisition and analysis of the data. AW wrote the article. BS, YG, CT, and YSL contributed to the critical revision of the manuscript. All authors read and approved the final manuscript.

## Funding

This work was supported by grant from the Ministry of Science and Technology of the People's Republic of China (2018YFC1311303), the Non-profit Central Research Institute Fund of Chinese Academy of Medical Sciences (2020-PT310-01), the 2021 Youth Talent Promotion Project in Henan Province to KL (Grant No. 2021HYTP054), and 2021 Joint Construction Project of Henan Medical Science and Technology Breakthrough Plan to KL (Grant No. LHGJ20210336).

## Conflict of interest

The authors declare that the research was conducted in the absence of any commercial or financial relationships that could be construed as a potential conflict of interest.

## Publisher's note

All claims expressed in this article are solely those of the authors and do not necessarily represent those of their affiliated organizations, or those of the publisher, the editors and the reviewers. Any product that may be evaluated in this article, or claim that may be made by its manufacturer, is not guaranteed or endorsed by the publisher.

## References

[1] GBD 2019 Stroke Collaborators. Global, regional, and national burden of stroke and its risk factors, 1990-2019: a systematic analysis for the Global Burden of Disease Study 2019. Lancet Neurol. (2021) 20:795–820. 10.1016/S1474-4422(21)00252-0 34487721 PMC8443449

[2] Baber U Mehran R Sartori S Schoos MM Sillesen H Muntendam P. Prevalence, impact, and predictive value of detecting subclinical coronary and carotid atherosclerosis in asymptomatic adults: the bioimage study. J Am Coll Cardiol. (2015) 65:1065–74. 10.1016/j.jacc.2015.01.017 25790876

[3] Kamtchum-Tatuene J Noubiap JJ Wilman AH Saqqur M Shuaib A Jickling GC. Prevalence of high-risk plaques and risk of stroke in patients with asymptomatic carotid stenosis: a meta-analysis. JAMA Neurol. (2020) 77:1524–35. 10.1001/jamaneurol.2020.2658 32744595 PMC7400201

[4] Saba L Saam T Jäger HR Yuan C Hatsukami TS Saloner D. Imaging biomarkers of vulnerable carotid plaques for stroke risk prediction and their potential clinical implications. Lancet Neurol. (2019) 18:559–72. 10.1016/S1474-4422(19)30035-3 30954372

[5] Kopczak A Schindler A Bayer-Karpinska A Koch ML Sepp D Zeller J. Complicated carotid artery plaques as a cause of cryptogenic stroke. J Am Coll Cardiol. (2020) 76:2212–22. 10.1016/j.jacc.2020.09.532 33153580

[6] Krist AH Davidson KW Mangione CM Barry MJ Cabana M Caughey AB. Screening for asymptomatic carotid artery stenosis: us preventive services task force recommendation statement. JAMA. (2021) 325:476–81. 10.1001/jama.2020.26988 33528542

[7] Sun L Clarke R Bennett D Guo Y Walters RG Hill M. Causal associations of blood lipids with risk of ischemic stroke and intracerebral hemorrhage in chinese adults. Nat Med. (2019) 25:569–74. 10.1038/s41591-019-0366-x 30858617 PMC6795549

[8] Holmes MV Millwood IY Kartsonaki C Hill MR Bennett DA Boxall R. Lipids, lipoproteins, and metabolites and risk of myocardial infarction and stroke. J Am Coll Cardiol. (2018) 71:620–32. 10.1016/j.jacc.2017.12.006 29420958 PMC5811927

[9] Di Angelantonio E Sarwar N Perry P Kaptoge S Ray KK Thompson A. Major lipids, apolipoproteins, and risk of vascular disease. JAMA. (2009) 302:1993–2000. 10.1001/jama.2009.1619 19903920 PMC3284229

[10] Carr SS Hooper AJ Sullivan DR Burnett JR. Non-HDL-cholesterol and apolipoprotein B compared with LDL-cholesterol in atherosclerotic cardiovascular disease risk assessment. Pathology. (2019) 51:148–54. 10.1016/j.pathol.2018.11.006 30595507

[11] Jacobson TA Ito MK Maki KC Orringer CE Bays HE Jones PH. National lipid association recommendations for patient-centered management of dyslipidemia: part 1–full report. J Clin Lipidol. (2015) 9:129–69. 10.1016/j.jacl.2015.02.003 25911072

[12] Di Bartolo BA Cartland SP Genner S Manuneedhi Cholan P Vellozzi M Rye KA. HDL improves cholesterol and glucose homeostasis and reduces atherosclerosis in diabetes-associated atherosclerosis. J Diabetes Res. (2021) 2021:6668506. 10.1155/2021/6668506 34095317 PMC8163542

[13] Grundy SM Stone NJ Bailey AL Beam C Birtcher KK Blumenthal RS. 2018 AHA/ACC/AACVPR/AAPA/ABC/ACPM/ADA/AGS/APHA/ASPC/NLA/PCNA guideline on the management of blood cholesterol: a report of the American College of Cardiology/American Heart Association Task Force on Clinical Practice Guidelines. Circulation. (2019) 139:e1082–143. 10.1161/CIR.0000000000000698 30586774 PMC7403606

[14] Badimón JJ Santos-Gallego CG Badimón L. [Importance of HDL cholesterol in atherothrombosis: how did we get here? Where are we going?]. Revista espanola de cardiologia. (2010) 63(Suppl 2):20–35. 10.1016/S0300-8932(10)70150-0 20540898

[15] Zhang N Hu X Zhang Q Bai P Cai M Zeng TS. Non-high-density lipoprotein cholesterol: high-density lipoprotein cholesterol ratio is an independent risk factor for diabetes mellitus: results from a population-based cohort study. J Diabetes. (2018) 10:708–14. 10.1111/1753-0407.12650 29437292

[16] Lin D Qi Y Huang C Wu M Wang C Li F. Associations of lipid parameters with insulin resistance and diabetes: a population-based study. Clin Nutr. (2018) 37:1423–9. 10.1016/j.clnu.2017.06.018 28673690

[17] Du T Yuan G Zhang M Zhou X Sun X Yu X. Clinical usefulness of lipid ratios, visceral adiposity indicators, and the triglycerides and glucose index as risk markers of insulin resistance. Cardiovasc Diabetol. (2014) 13:146. 10.1186/s12933-014-0146-3 25326814 PMC4209231

[18] Yang S Zhong J Ye M Miao L Lu G Xu C. Association between the non-HDL-cholesterol to HDL-cholesterol ratio and non-alcoholic fatty liver disease in chinese children and adolescents: a large single-center cross-sectional study. Lipids Health Dis. (2020) 19:242. 10.1186/s12944-020-01421-5 33222696 PMC7681973

[19] Wang K Shan S Zheng H Zhao X Chen C Liu C. Non-HDL-cholesterol to HDL-cholesterol ratio is a better predictor of new-onset non-alcoholic fatty liver disease than non-HDL-cholesterol: a cohort study. Lipids Health Dis. (2018) 17:196. 10.1186/s12944-018-0848-8 30131058 PMC6104008

[20] Kim SW Jee JH Kim HJ Jin SM Suh S Bae JC. Non-HDL-cholesterol/HDL-cholesterol is a better predictor of metabolic syndrome and insulin resistance than apolipoprotein B/apolipoprotein A1. Int J Cardiol. (2013) 168:2678–83. 10.1016/j.ijcard.2013.03.027 23545148

[21] Qin G Tu J Zhang C Tang X Luo L Wu J. The value of the apoB/apoA? ratio and the non-HDL-C/HDL-C ratio in predicting carotid atherosclerosis among chinese individuals with metabolic syndrome: a cross-sectional study. Lipids Health Dis. (2015) 14:24. 10.1186/s12944-015-0023-4 25885111 PMC4399243

[22] Masson W Epstein T Huerín M Lobo M Molinero G Siniawski D. Association between non-HDL-C/HDL-C ratio and carotid atherosclerosis in postmenopausal middle-aged women. Climacteric. (2019) 22:518–22. 10.1080/13697137.2019.1631787 31287342

[23] Liu Y Zhang Z Xia B Wang L Zhang H Zhu Y. Relationship between the non-HDLC-to-HDLC ratio and carotid plaques in a high stroke risk population: a cross-sectional study in China. Lipids Health Dis. (2020) 19:168. 10.1186/s12944-020-01344-1 32660519 PMC7359500

[24] Virani SS Catellier DJ Pompeii LA Nambi V Hoogeveen RC Wasserman BA. Relation of cholesterol and lipoprotein parameters with carotid artery plaque characteristics: the atherosclerosis risk in communities (ARIC) carotid MRI Study. Atherosclerosis. (2011) 219:596–602. 10.1016/j.atherosclerosis.2011.08.001 21868017 PMC3226845

[25] Nidorf SM Fiolet A Abela GS. Viewing atherosclerosis through a crystal lens: how the evolving structure of cholesterol crystals in atherosclerotic plaque alters its stability. J Clin Lipidol. (2020) 14:619–30. 10.1016/j.jacl.2020.07.003 32792218

[26] Zhu L Lu Z Zhu L Ouyang X Yang Y He W. Lipoprotein ratios are better than conventional lipid parameters in predicting coronary heart disease in Chinese Han people. Kardiologia Polska. (2015) 73:931–8. 10.5603/KP.a2015.0086 25985729

[27] Kouvari M Panagiotakos DB Chrysohoou C Georgousopoulou EN Tousoulis D Pitsavos AC. Sex-related differences of the effect of lipoproteins and apolipoproteins on 10-year cardiovascular disease risk; insights from the Attica Study (2002-2012). Molecules. (2020) 25:1506. 10.3390/molecules25071506 32225033 PMC7180686

[28] Touboul PJ Hennerici MG Meairs S Adams H Amarenco P Bornstein N. Mannheim carotid intima-media thickness and plaque consensus (2004-2006-2011). In: An Update on Behalf of the Advisory Board of the 3rd, 4th and 5th Watching the Risk Symposia, at the 13th, 15th and 20th European Stroke Conferences. Mannheim; Brussels; Hamburg (2012). p. 290–6. 10.1159/000343145 PMC3760791 23128470

[29] Touboul PJ Hennerici MG Meairs S Adams H Amarenco P Desvarieux M. Mannheim intima-media thickness consensus. Cerebrovasc Dis. (2004) 18:346–9. 10.1159/000081812 15523176

[30] Sztajzel R. Ultrasonographic assessment of the morphological characteristics of the carotid plaque. Swiss Med Weekly. (2005) 135:635–43. 16380850 10.4414/smw.2005.11038

[31] Poredoš P Cevc M Blinc A. Characteristics of atherosclerosis in femoropopliteal artery and its clinical relevance. Atherosclerosis. (2021) 335:31–40. 10.1016/j.atherosclerosis.2021.09.012 34547588

[32] Song P Fang Z Wang H Cai Y Rahimi K Zhu Y. Global and regional prevalence, burden, and risk factors for carotid atherosclerosis: a systematic review, meta-analysis, and modelling study. Lancet Global Health. (2020) 8:e721–9. 10.1016/S2214-109X(20)30117-0 32353319

[33] Ooi YC Gonzalez NR. Management of extracranial carotid artery disease. Cardiol Clin. (2015) 33:1–35. 10.1016/j.ccl.2014.09.001 25439328 PMC4694631

[34] Naylor AR Ricco JB de Borst GJ Debus S de Haro J Halliday A. Editor's choice - management of atherosclerotic carotid and vertebral artery disease: 2017 Clinical Practice Guidelines of the European Society for Vascular Surgery (ESVS). Eur J Vasc Endovasc Surg. (2018) 55:3–81. 10.1016/j.ejvs.2017.10.014 28851594

[35] Saba L Brinjikji W Spence JD Wintermark M Castillo M Borst GJD. Roadmap consensus on carotid artery plaque imaging and impact on therapy strategies and guidelines: an international, multispecialty, expert review and position statement. Am J Neuroradiol. (2021) 42:1566–75. 10.3174/ajnr.A7223 34326105 PMC8423069

[36] Willeit P Tschiderer L Allara E Reuber K Seekircher L Gao L. Carotid intima-media thickness progression as surrogate marker for cardiovascular risk: meta-analysis of 119 clinical trials involving 100 667 patients. Circulation. (2020) 142:621–42. 10.1161/CIRCULATIONAHA.120.046361 32546049 PMC7115957

[37] Wu J Zhang J Wang A Chen S Wu S Zhao X. Association between non-high-density lipoprotein cholesterol levels and asymptomatic vulnerable carotid atherosclerotic plaques. Eur J Neurol. (2019) 26:1433–8. 10.1111/ene.13973 31002203

[38] Expert Expert Panel on Detection Evaluation and and Treatment of High Blood Cholesterol in Adults. Executive summary of the third report of the National Cholesterol Education Program (NCEP) expert panel on detection, evaluation, and treatment of high blood cholesterol in adults (adult treatment Panel III). JAMA. (2001) 285:2486–97. 10.1001/jama.285.19.2486 11368702

[39] Mach F Baigent C Catapano AL Koskinas KC Casula M Badimon L. 2019 ESC/EAS guidelines for the management of dyslipidaemias: lipid modification to reduce cardiovascular risk. Atherosclerosis. (2019) 290:140–205. 10.1093/eurheartj/ehz455 31591002

[40] Santos-Gallego CG Rosenson RS. Role of HDL in those with diabetes. Curr Cardiol Rep. (2014) 16:512. 10.1007/s11886-014-0512-5 25199216

[41] Santos-Gallego CG Giannarelli C Badimón JJ. Experimental models for the investigation of high-density lipoprotein-mediated cholesterol efflux. Curr Atheroscler Rep. (2011) 13:266–76. 10.1007/s11883-011-0177-0 21484293

